# Statistics of natural scene structures and scene categorization

**DOI:** 10.1186/1471-2202-13-S1-P7

**Published:** 2012-07-16

**Authors:** Xin Chen, Weibing Wan, Zhiyong Yong

**Affiliations:** 1 Brain and Behavior Discovery Institute; 2 Department of Ophthalmology; 3 Vision Discovery Institute, Georgia Health Sciences University, Augusta, Georgia, 30912, USA

## 

Humans can grasp the gist of complex natural scenes very quickly and can remember extraordinarily rich details in thousands of scenes viewed for a very brief period [1,2]. This amazing ability of rapid scene perception challenges both the traditional view of image-based, bottom-up visual processing [3] and recent models of scene categorization based on global visual features and features at low spatial frequency [4]. Low-level visual features such as edges, junctions, and various image gradients are insufficient for revealing the content of complex natural scenes. On the other hand, global visual features and features at low spatial frequency cannot encode the extraordinarily rich spatial concatenations of visual features in natural scenes.

We proposed natural scene structures, i.e., multi-size, multi-scale, spatial concatenations of visual features, as the basic encoding units of natural scenes and scene categories. Natural scene structures convey various amount of information about scene identities and categories since general structures are shared by more scenes while specific structures are shared by only a few scenes. Thus, any natural scene and category can be represented by a probability distribution based on a set of natural scene structures and their spatial concatenations. These structural representations are robust against variations due to noises, occlusions, changes in scales, and other factors and require no isolation of objects or figure-background segmentation, nor computation of global scene features.

To test this model of natural scenes, we compiled a large set of natural scene structures from a database of natural scenes and examined the information conveyed by the natural scene structures about natural scene categories. We then selected a set of natural scene structures with high information content, organized them into clusters, and developed a probabilistic model on the clusters of selected scene structures for each scene category. Finally, we categorized natural scenes by performing Bayesian inference based on these probability distributions. We found that the model achieved a high performance of categorization on a large dataset of natural scene categorizes. We also tested this model of natural scenes using human psychophysics. We constructed experimental stimuli that consisted of only the selected natural scene structures and asked human subjects to perform scene categorization. We either maintained or shuffled the spatial locations of the natural scene structures in the experimental stimuli. We found that the subject performance was significantly above chance even when the selected scene structures covered only a small portion of the scenes. Furthermore, shuffling the spatial locations of the scene structures significantly reduced the subject performance. These results support our statistical model of natural scenes using natural scene structures as encoding units.
